# 中国Castleman病诊断与治疗指南（2025年版）

**DOI:** 10.3760/cma.j.cn121090-20250101-00001

**Published:** 2025-03

**Authors:** 中华医学会血液学分会罕见病学组 中国Castleman病协作组

## Abstract

2021年，中国Castleman病协作组发布了《中国Castleman病诊断与治疗专家共识（2021年版）》。近年来，Castleman病诊疗领域取得了较大的进展。基于近年来最新研究成果，经过协作组专家共同商讨，在前述共识的基础上，根据牛津证据分级系统制定了本指南。

## Abstract (en)

In 2021, China Castleman Disease Network（CDCN）published the the first consensus of the diagnosis and treatment of Castleman disease in China（2021）. In recent years, significant progress has been made in the field of Castleman disease. Based on the most up-to-date research findings and through collective discussion of CDCN experts, the new guideline is developed according to the Oxford Center for Evidence-Based Medicine（OCEBM）levels of evidence system, building upon the aforementioned consensus.

Castleman病（Castleman disease, CD）是一种纳入我国《第一批罕见病目录》的罕见血液病，因发现者为Benjamin Castleman而得名[1]。2021年，中国Castleman病协作组（China Castleman Disease Network，CCDN）成立，并发布了《中国Castleman病诊断与治疗专家共识（2021年版）》[2]。近年来，国内外在该领域的研究有较大进展。经CCDN专家共同商讨，纳入近年来的研究成果，在前述国内共识的基础上制定本指南，以供临床参考。本指南已在国际实践指南注册与透明化平台（Practice guideline REgistration for transPAREncy, PREPARE）注册，注册号为PREPARE-2024CN510。

根据牛津证据分级系统（Oxford Center for Evidence-Based Medicine，OCEBM）对证据质量和推荐强度进行分级（表1）[3]。

**表1:** 基于牛津证据分级系统（OCEBM）的证据及推荐强度等级

推荐强度	证据级别	描述
A	1a	同质性较好的多项随机对照研究（RCT）的系统评价
	1b	结果可信区间小的单项RCT
	1c	显示“全或无效应”的任何证据
B	2a	同质性较好的队列研究的系统评价
	2b	单个的队列研究（包括低质量的RCT）
	2c	基于患者结局的研究
	3a	同质性较好的病例对照研究的系统评价
	3b	单个病例对照研究
C	4	病例系列报告，低质量队列研究和病例对照研究
D	5	专家意见（基于经验，未经严格论证）

一、定义

CD是一组具有特征性组织病理学表现的罕见淋巴组织增殖性疾病，淋巴结病理是诊断该病的金标准[2],[4]。推荐对病变淋巴结行完整或部分切除活检，深部或难以切除的病灶亦可行空芯针穿刺活检。从病理形态上，CD可进一步分为透明血管型（又称玻璃样血管型）CD（hyaline vascular subtype of CD, HV-CD）、浆细胞型CD（plasma cell subtype of CD, PC-CD）及混合型CD（mixed type of CD）[2]。不同病理亚型的镜下表现[2]：①HV-CD：淋巴结体积通常较大（数厘米至十余厘米），有完整包膜和丰富血供。镜下形态改变主要包括淋巴滤泡增多、生发中心缩小、套细胞区增宽及滤泡间区血管增生。萎缩的生发中心淋巴细胞削减，仅剩余显著的滤泡树突细胞成分，增生的套细胞可呈同心圆状排列或出现“洋葱皮”样外观，部分滤泡内可有多个萎缩的生发中心。滤泡间区淋巴窦消失，多有显著性厚壁小血管增生，且血管壁可出现程度不等的玻璃样变性。部分玻璃样变性的小血管还垂直长入生发中心形成“棒棒糖”样外观。淋巴结包膜和小梁也多有增厚、增宽伴玻璃样变性。②PC-CD：肿大淋巴结的体积通常较小。镜下可见HV-CD样淋巴滤泡，但部分病例或部分病灶的滤泡生发中心萎缩不明显，甚至会出现生发中心增生和扩大，伴数量显著增多的浆细胞浸润，部分病例可表现为滤泡间区弥漫性、致密的浆细胞增生并完全取代滤泡间区正常结构。多数病例浆细胞形态成熟，无免疫球蛋白轻链限制性表达。③混合型CD：形态特点兼具HV-CD及PC-CD的特征，可理解为两者的过渡形态或组合形式。

值得指出的是，淋巴结病理形态符合CD的患者，需要首先除外可能引起淋巴结Castleman样改变的其他相关疾病，包括（但不限于）感染性疾病（如HIV、梅毒、EB病毒感染，结核等）、肿瘤性疾病（如POEMS综合征、淋巴瘤、滤泡树突细胞肉瘤、浆细胞瘤等）、自身免疫性疾病（如系统性红斑狼疮、类风湿关节炎、自身免疫性淋巴细胞增生综合征等）[4]。对于前述疾病所致的淋巴结Castleman样改变，治疗上应以对应基础疾病为主，故不是本指南讨论的重点。

二、CD的临床分型

对于淋巴结病理符合CD表现的患者，除外前述可能引起淋巴结CD样改变的基础疾病后，根据淋巴结受累区域的不同，可以从临床角度分为单中心型（unicentric CD, UCD）和多中心型（multicentric CD, MCD）。对于后者，根据是否感染人类疱疹病毒8型（HHV-8）可进一步分为HHV-8阳性MCD及HHV-8阴性MCD[4]（图1）。

**图1:**
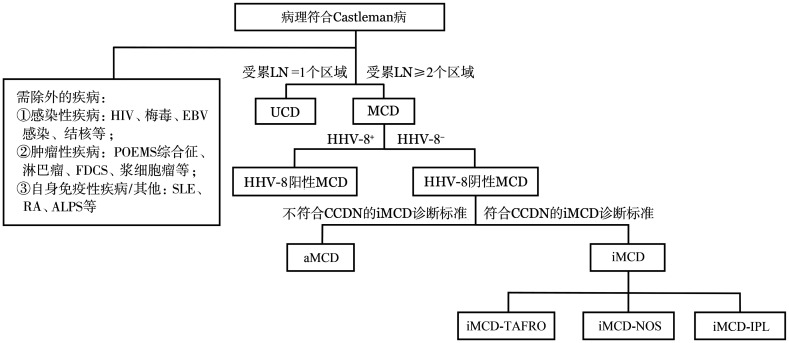
Castleman病的临床分型[2] **注** HIV：人类免疫缺陷病毒；EBV：EB病毒；FDCS：滤泡树突细胞肉瘤；SLE：系统性红斑狼疮；RA：类风湿关节炎；ALPS：自身免疫性淋巴细胞增生综合征；LN：淋巴结；UCD：单中心型Castleman病；MCD：多中心型Castleman病；HHV-8：人类疱疹病毒8型；CCDN：中国Castleman病协作组；iMCD：特发性MCD；aMCD：无症状性MCD；iMCD-TAFRO：iMCD-TAFRO综合征；iMCD-NOS：iMCD-非特指型；iMCD-IPL：iMCD-特发性浆细胞性淋巴结病

UCD：累及单个淋巴结区域，多数患者除淋巴结肿大外无全身症状。少数患者可伴有与MCD类似的高炎症表现[5]，也有部分患者合并副肿瘤天疱疮、闭塞性细支气管炎、血清淀粉样蛋白A型（AA）淀粉样变[6]–[8]，并同时存在上述疾病相关临床表现。

MCD：有多个（≥2个）淋巴结区域受累（受累淋巴结短径≥1 cm）的CD患者被归为MCD。根据HHV-8感染情况，MCD可分为HHV-8阳性MCD和HHV-8阴性MCD。前者在国内患者中所占比例低（国内回顾性研究中仅占所有MCD的1.6％[9]），对利妥昔单抗治疗反应好[10]。后者若满足特发性MCD（idiopathic MCD, iMCD）的诊断标准[2]，则诊断为iMCD。对于不存在症状和高炎症状态，从而不满足iMCD诊断标准的MCD患者，则诊断为无症状性MCD（asymptomatic MCD, aMCD）[2],[9]。对于iMCD患者，还可根据临床表现进一步分为iMCD-TAFRO亚型[11]、iMCD-IPL（idiopathic plasmacytic lymphadenopathy）亚型[12]和iMCD-NOS（not otherwise specified）亚型。

病理分型和临床分型的关系：在临床分型为UCD的患者中，虽然透明血管型的比例较高（70％～90％），但也有10％～30％的UCD患者病理类型为浆细胞型或混合型，而此类UCD患者更易出现与iMCD类似的高炎症表现。MCD患者中浆细胞型和混合型的比例较高，透明血管型、浆细胞型和混合型MCD患者的比例分别为21％、39％和39％[13]。此外，对于特定的MCD亚型，病理分型的分布也有相应特点，如iMCD-IPL亚型患者对应的病理分型仅有浆细胞型和混合型[12]。

三、CD的诊断标准

排除前文所述可能引起淋巴结Castleman样改变的基础疾病后：

UCD：病理符合CD，且仅有单个淋巴结区域受累[6]；

MCD：病理符合CD，存在≥2个淋巴结区域受累（淋巴结短径需≥1 cm）。对于MCD患者，推荐采用外周血HHV-8 DNA检测和（或）淋巴结病理LANA-1（latency-associated nuclear antigen-1）免疫组化染色判断HHV-8状态，区分为HHV-8阳性和HHV-8阴性MCD。

iMCD：对于HHV-8阴性MCD，若符合以下两条主要标准+至少两条次要标准（其中至少一条是实验室标准），且排除可能引起淋巴结Castleman样改变的基础疾病后，可诊断为iMCD[2]。主要标准：①淋巴结病理符合CD；②存在≥2个淋巴结区域受累（淋巴结短径≥1 cm）。次要标准：分为实验室标准和临床标准。实验室标准包括：①C反应蛋白（CRP）>10 mg/L或红细胞沉降率>20 mm/1 h（女性）或15 mm/1 h（男性）；②贫血（HGB<100 g/L）；③血小板减少（PLT<100×10^9^/L）或增多（PLT>350×10^9^/L）；④血清白蛋白<35 g/L；⑤估算肾小球滤过率（eGFR）<60 ml·min^−1^·（1.73 m^2^）^−1^或蛋白尿（尿总蛋白>150 mg/24 h或100 mg/L）；⑥血清IgG>17 g/L。临床标准包括：①全身症状：盗汗、发热（体温>38 °C）、体重下降（6个月下降≥10％）或乏力（影响工具性日常生活活动）；②肝大和（或）脾大；③水肿或浆膜腔积液；④皮肤樱桃血管瘤或紫罗兰样丘疹；⑤淋巴细胞性间质性肺炎。

iMCD-TAFRO：符合iMCD诊断的前提下，满足以下所有主要标准和≥1个次要标准[11]。主要标准：①血小板减少（治疗前PLT最低水平<100×10^9^/L）；②水肿或浆膜腔积液；③发热或高炎症状态：原因不明的发热（体温>37.5 °C）或CRP>20 mg/L；④器官肿大：包括≥2个淋巴结区域的轻度淋巴结肿大、肝大和（或）脾大。次要标准：①肾功能不全：eGFR<60 ml·min^−1^·（1.73 m^2^）^−1^，肌酐>97 µmol/L（女性）或>115 µmol/L（男性），或肾功能不全需血液透析治疗；②TAFRO相关骨髓改变：骨髓纤维化或骨髓巨核细胞增生。

iMCD-IPL：符合iMCD诊断标准且不符合iMCD-TAFRO，同时满足下述3条标准[12]：①外周血IgG>17.4 g/L或超过正常上限；②淋巴结病理类型为浆细胞型或混合型；③血小板增多（PLT>350×10^9^/L）。

iMCD-NOS：符合iMCD诊断标准，且不符合iMCD-TAFRO或iMCD-IPL诊断标准。

四、CD的治疗

1. 治疗前评估：启动治疗前需对患者进行全面评估，相关的检查至少应包括：①病理检查：有经验的病理科医师评估/复核病理（参考本指南病理部分）；②症状评估：评价有无发热、疲乏、厌食、体重下降、呼吸困难、皮疹、浆膜腔积液等相关症状；评价有无肿瘤压迫相关症状；③炎症状态及器官损伤评估：血常规、尿常规、肝肾功能、红细胞沉降率、CRP、血清白蛋白、乳酸脱氢酶、白细胞介素-6（IL-6）、肺功能（通气+弥散，对于存在呼吸困难症状的患者）；④影像学检查：颈胸腹盆（增强）CT检查或全身PET-CT检查；⑤鉴别诊断相关检查：病原学检测（HIV抗体及抗原、EB病毒DNA、梅毒抗体、HHV-8 DNA）、免疫相关检测（抗核抗体谱、类风湿因子、免疫球蛋白定量、IgG4）、M蛋白相关检测（血清蛋白电泳、血尿免疫固定电泳）。

2. UCD的治疗：完整手术切除病灶是UCD最核心的治疗方式[6]，对于所有UCD患者（无论是否合并高炎症状态、副肿瘤天疱疮、闭塞性细支气管炎或AA淀粉样变），都应该首先评估病灶的可切除性。若能完整手术切除病灶，无论患者是否合并全身症状，都首选完整切除病灶（证据等级2a，B级推荐）。对于难以完整手术切除病灶的患者：①无症状：观察（证据等级2b，B级推荐）；②压迫症状：利妥昔单抗±糖皮质激素或利妥昔单抗±化疗（证据等级2b，B级推荐）；③伴有高炎症状态：司妥昔单抗（证据等级2b，B级推荐）或参考针对iMCD的其他方案进行治疗（证据等级4，C级推荐）。前述治疗后，需再次评估完整手术切除的可能性，对于治疗后具备可切除性的患者，仍推荐完整手术切除病灶（证据等级2a，B级推荐）。对于治疗后仍难以手术，且依旧存在症状的患者，可考虑放疗（证据等级4，C级推荐）。动脉栓塞可在手术切除前用于血供丰富的病灶，以增加手术的安全性和成功率；也可用于难以手术切除、经药物治疗后症状持续的患者（证据等级4，C级推荐）。

3. MCD的整体治疗原则：对于HHV-8阳性MCD，推荐利妥昔单抗为基础的方案（如利妥昔单抗±脂质体阿霉素/阿霉素±糖皮质激素）[2],[14]–[15]（证据等级2a，B级推荐），若患者同时合并HIV感染，还需要请相关科室协助制定抗HIV治疗方案。对于HHV-8阴性MCD，无症状且不符合iMCD诊断标准的aMCD患者可暂观察，待符合iMCD诊断标准后参考iMCD进行治疗。对于iMCD患者，依据国际Castleman病协作网络（Castleman Disease Collaborative Network，CDCN）危险度分层定义的“非重型”和“重型”采取不同的治疗策略[16]。对于iMCD患者，符合下述5条标准中2条及以上则考虑重型iMCD，否则为非重型iMCD：①美国东部肿瘤协作组评分≥2分；②eGFR<30 ml·min^−1^·（1.73 m^2^）^−1^；③重度水肿和（或）腹水、胸水、心包积液；④HGB≤80 g/L；⑤肺部受累或伴气促的间质性肺炎。由于iMCD是一种缺乏高级别循证医学证据的罕见病，初治或难治/复发的“非重型”和“重型”iMCD患者，均推荐积极参与设计良好的临床研究。

4. iMCD的治疗（图2）：①非重型iMCD的一线治疗：推荐的方案包括司妥昔单抗±泼尼松（证据等级1b，A级推荐）[17]–[18]，沙利度胺+环磷酰胺+泼尼松（TCP）方案（证据等级2b，B级推荐）[19]，硼替佐米+环磷酰胺+地塞米松（BCD）方案（证据等级2b，B级推荐）[20]，托珠单抗±泼尼松（证据级别2b，B级推荐）[21]，利妥昔单抗联合环磷酰胺+长春新碱+泼尼松（R-CVP）方案或利妥昔单抗±泼尼松（R±P）方案（证据等级3a，B级推荐）[16],[22]–[23]。②重型iMCD的一线治疗：推荐的一线方案包括司妥昔单抗联合大剂量糖皮质激素冲击治疗[16]（证据等级1b，A级推荐），BCD方案（证据等级2b，B级推荐）[20]，托珠单抗联合大剂量糖皮质激素冲击治疗[16]（证据级别2b，B级推荐），司妥昔单抗联合BCD方案（证据等级3b，B级推荐）[24]，利妥昔单抗+硼替佐米+地塞米松（RVD）方案（证据等级4，C级推荐）[25]。以上方案的具体药物应用见表2。③iMCD治疗后的疗效评价：推荐采用CDCN疗效评估标准[16]（表3[2]），从症状改善、生化疗效、淋巴结缩小三个维度进行评估。司妥昔单抗治疗后，不采用IL-6水平作为疗效评估的依据。④iMCD的二线及后线治疗：经前线治疗后未达到缓解的患者，或达到缓解后再次疾病进展的患者，需考虑后线治疗（图2）。后线治疗可以首先选择未使用过的前述一线治疗方案，如既往未接受过司妥昔单抗的患者可考虑以该药为基础的治疗（证据等级1b，A级推荐）。也可选择包括西罗莫司（证据等级3a，B级推荐）[26]–[27]、联合化疗（证据等级3a，B级推荐）[28]–[29]等的后线治疗方案。此外，对于多线治疗后仍难治的患者，还可考虑来那度胺、芦可替尼、奥布替尼等潜在治疗方案。⑤iMCD-TAFRO亚型的治疗：总体原则仍参考iMCD，按“重型”和“非重型”进行一线治疗选择，BCD方案是此类患者较为合适的治疗选择（推荐等级2b，B级推荐）[20]。此外，既往文献中环孢素被认为对iMCD-TAFRO有效，尤其是对于改善腹水和血小板降低[16]。

**图2:**
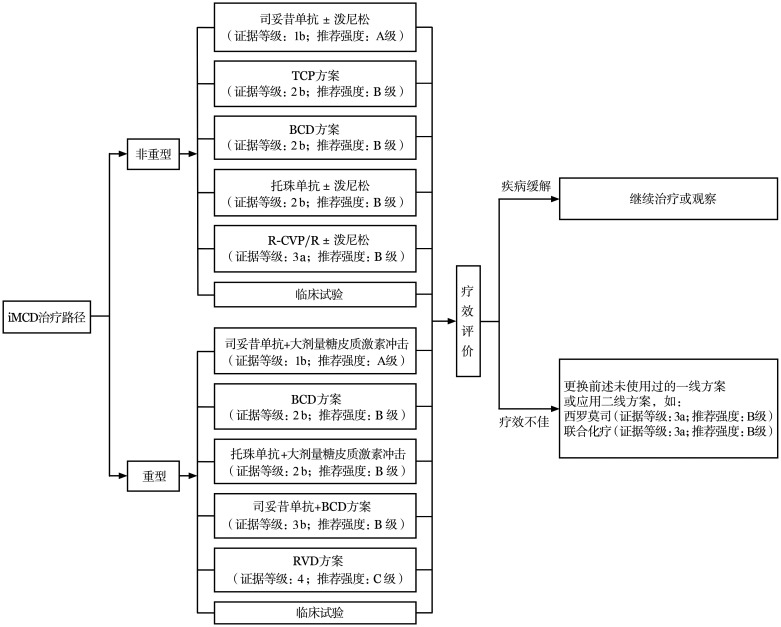
特发性多中心型Castleman病的治疗 **注** iMCD：特发性多中心型Castleman病；TCP：沙利度胺+环磷酰胺+泼尼松；BCD：硼替佐米+环磷酰胺+地塞米松；R-CVP：利妥昔单抗+环磷酰胺+长春新碱+泼尼松；R：利妥昔单抗；RVD：利妥昔单抗+硼替佐米+地塞米松

**表2:** 针对特发性多中心型Castleman病（iMCD）的治疗方案

方案名称	具体用法用量
司妥昔单抗±泼尼松	司妥昔单抗11 mg/kg每3周1次静脉给药，治疗有效患者长期用药，直至疾病进展或不耐受，达到缓解后可酌情将给药间隔延长至6周；泼尼松1 mg·kg^−1^·d^−1^起始，4～8周后逐渐减量并停用
沙利度胺+环磷酰胺+泼尼松（TCP）	沙利度胺100 mg每晚1次口服，环磷酰胺300 mg/m^2^每周1次口服，泼尼松1 mg/kg每周2次口服，治疗有效患者用药满1年后改为沙利度胺单药维持治疗1年
硼替佐米+环磷酰胺+地塞米松（BCD）	每4周为1个疗程，硼替佐米1.3 mg/m^2^，每周1次，皮下注射；环磷酰胺300 mg/m^2^，每周1次，口服；地塞米松40 mg，每周1次，口服。治疗9个疗程后调整为BD方案（硼替佐米1.3 mg/m^2^，每2周1次，皮下注射；地塞米松20 mg，每2周1次，口服）维持治疗一年
托珠单抗±泼尼松	托珠单抗8 mg/kg每2周1次静脉给药，治疗有效患者长期用药，直至疾病进展或不耐受；泼尼松1 mg·kg^−1^·d^−1^起始，4～8周后逐渐减量并停用
利妥昔单抗+环磷酰胺+长春新碱+泼尼松（R-CVP）	每3～4周为1个疗程，利妥昔单抗375 mg/m^2^，第1天，静脉输注；环磷酰胺750 mg/m^2^，第1天，静脉输注；长春新碱1.4 mg/m^2^（总剂量不超过2 mg），第1天，静脉输注；泼尼松100 mg/d，第1～5天，口服，4～6个疗程
利妥昔单抗（R）±泼尼松	每4周为1个疗程，利妥昔单抗375 mg/m^2^，第1天，静脉输注；泼尼松1 mg·kg^−1^·d^−1^，第1～7天，0.5 mg·kg^−1^·d^−1^，第8～14天，口服
司妥昔单抗+大剂量糖皮质激素冲击	司妥昔单抗11 mg/kg每3周1次静脉给药，联合甲泼尼龙500 mg，每日1次，静脉输注，3～5 d。为了迅速起效，用药第1个月时可将司妥昔单抗调整为每周1次，之后调整为每3周1次静脉输注
托珠单抗+大剂量糖皮质激素冲击	托珠单抗8 mg/kg每2周1次静脉给药，联合甲泼尼龙500 mg每日1次，静脉输注，3～5 d
司妥昔单抗+BCD	BCD方案同前，早期联用司妥昔单抗（对于细胞因子风暴重、脏器损伤明显患者，早期司妥昔单抗每周1次给药）
利妥昔单抗+硼替佐米+地塞米松（RVD）	每4周为1个疗程，利妥昔单抗375 mg/m^2^，第1天，静脉输注；硼替佐米1.3 mg/m^2^，每周1次，皮下注射；地塞米松40 mg，每周1次，口服。4个疗程后达到缓解的患者可暂停药物观察
西罗莫司	单药口服，1 mg/d起始（范围1～7 mg/d），目标谷浓度5～15 ng/ml
联合化疗	VDT-ACE-R（硼替佐米+地塞米松+沙利度胺+阿霉素+环磷酰胺+依托泊苷+利妥昔单抗），R-CHOP（利妥昔单抗+环磷酰胺+阿霉素+长春新碱+泼尼松）

**注** 以上方案中，疗程及剂量推荐主要来自成年人群数据，儿童可能需要酌情调整

**表3:** 特发性多中心型Castleman病的疗效评估标准[2]

整体疗效	生化疗效	淋巴结（根据Cheson标准）	症状改善^e^
CR^a^	CRP、血红蛋白、白蛋白、GFR恢复正常^f^	CR	恢复至基线（发病前）
PR^b^	CRP、血红蛋白、白蛋白、GFR均改善>50％	PR	4个症状（疲乏、厌食、发热、体重下降）均改善，但未恢复至发病前
SD^c^	CRP、血红蛋白、白蛋白、GFR均改善<50％，或恶化<25％	未达PR或CR	4个症状（疲乏、厌食、发热、体重下降）中至少1个症状（但不是所有症状）改善
PD^d^	CRP、血红蛋白、白蛋白、GFR中任一项恶化>25％	增大>25％	≥2次评估提示4个症状（疲乏、厌食、发热、体重下降）中任一症状恶化^g^

**注** CR：完全缓解；PR：部分缓解；SD：疾病稳定；PD：疾病进展；CRP：C反应蛋白；GFR：肌酐清除率；^a^指生化疗效、淋巴结、症状改善均达CR；^b^指生化疗效、淋巴结、症状改善均≥PR；^c^指生化疗效、淋巴结、症状改善均未达到PD且不符合PR或CR标准；^d^指生化疗效、淋巴结、症状中任何一项PD；^e^指疲乏或厌食的通用毒性标准（common toxicity criteria，CTC）级别较治疗前下降≥1级，发热体温较治疗前下降≥1 °C，体重较治疗前增加≥5％；^f^指CRP≤10 mg/L，HGB≥130 g/L（男）或115 g/L（女），白蛋白≥35 g/L，GFR≥60 ml·min^−1^·（1.73 m^2^）^−1^；^g^指CTC级别较治疗前恶化≥1级

四、随访

1. UCD：对于已经手术完整切除病灶且已无症状的UCD患者，术后3个月进行查体、生化检测（血常规、肝肾功能、白蛋白、CRP、免疫球蛋白等）及CT检查，之后每年完成查体、生化及CT检查[6]；对于尚未完整切除病灶及仍然存在症状和高炎症状态的患者，需酌情密切观察病情变化。合并副肿瘤天疱疮、闭塞性细支气管炎的UCD患者预后较差，需要密切观察皮疹和肺功能情况。

2. MCD：aMCD患者诊断后，起初需每3个月完成症状询问、查体、生化检测（血常规、肝肾功能、白蛋白、CRP、免疫球蛋白等）及CT检查，若病情平稳，后续随访间隔可拉长至6～12个月，注意是否转化为iMCD。iMCD患者接受全身治疗后，治疗初期根据病情密切监测；疾病相对稳定后，除每3个月进行症状、查体及血液生化评估外，每3个月随诊CT等影像学检查直至获得最佳疗效，之后影像学检查复查的间隔可以拉长至6～12个月[16]。
